# Oxidation of Hepatic Carnitine Palmitoyl Transferase-I (CPT-I) Impairs Fatty Acid Beta-Oxidation in Rats Fed a Methionine-Choline Deficient Diet

**DOI:** 10.1371/journal.pone.0024084

**Published:** 2011-09-01

**Authors:** Gaetano Serviddio, Anna M. Giudetti, Francesco Bellanti, Paola Priore, Tiziana Rollo, Rosanna Tamborra, Luisa Siculella, Gianluigi Vendemiale, Emanuele Altomare, Gabriele V. Gnoni

**Affiliations:** 1 Centre for the Study of Liver Diseases, Institute of Internal Medicine, Department of Medical and Occupational Sciences, University of Foggia, Foggia, Italy; 2 Laboratory of Biochemistry and Molecular Biology, Department of Biological and Environmental Sciences and Technologies, University of Salento, Lecce, Italy; 3 IRCCS-Casa Sollievo della Sofferenza, San Giovanni Rotondo, Foggia, Italy; Sapienza University of Rome, Italy

## Abstract

There is growing evidence that mitochondrial dysfunction, and more specifically fatty acid β-oxidation impairment, is involved in the pathophysiology of non-alcoholic steatohepatitis (NASH). The goal of the present study was to achieve more understanding on the modification/s of carnitinepalmitoyltransferase-I (CPT-I), the rate-limiting enzyme of the mitochondrial fatty acid β-oxidation, during steatohepatitis. A high fat/methionine-choline deficient (MCD) diet, administered for 4 weeks, was used to induce NASH in rats. We demonstrated that CPT-Iactivity decreased, to the same extent, both in isolated liver mitochondria and in digitonin-permeabilized hepatocytes from MCD-diet fed rats. At the same time, the rate of total fatty acid oxidation to CO_2_ and ketone bodies, measured in isolated hepatocytes, was significantly lowered in treated animals when compared to controls. Finally, an increase in CPT-I mRNA abundance and protein content, together with a high level of CPT-I protein oxidation was observed in treated rats. A posttranslational modification of rat CPT-I during steatohepatitis has been here discussed.

## Introduction

The term non-alcoholic steatohepatitis (NASH) describes histopathological findings typical of alcoholic liver disease in a group of patients without significant alcohol consumption [1]. NASH is observed in a subset of patients with non-alcoholic fatty liver disease (NAFLD), a pathology comprising a wide spectrum of liver damage, ranging from simple macrovescicular steatosis to steatohepatitis, advanced fibrosis, and cirrhosis [1]. The clinical relevance of these conditions is related to the high prevalence of NAFLD in the population and to the possible evolution of NASH towards end-stage liver disease, including hepatocellular carcinoma, as well as the need for liver transplantation [2]. Even though the mechanisms of the progression from simple steatosis to NASH are not completely understood, mitochondrial dysfunction has been proposed as a key factor. Indeed, ultrastructural alterations, impairment of ATP synthesis and increased production of reactive oxygen species (ROS) have been reported in liver mitochondria from NASH human patients and from rodent model for this pathology [3]–[8].

Some of us have recently described an altered hepatic mitochondrial function in rats affected by NASH induced by a methionine-choline deficient (MCD) diet [4]. Rodents fed a MCD diet develop a steatohepatitis producing hepatic lesions and changes in liver redox balance, mimicking the impairment observed in patients with NASH [9], [10]. Fat accumulation enhances mitochondrial ROS production [11] which, in turn, may cause oxidative stress. The most important cellular damage caused by ROS is peroxidation of membrane lipids resulting in generalized alteration of the membrane function [12]. Lipid peroxidation products can react with functional groups of amino acids in proteins and enzymes to form adducts that may alter protein function [13]. This has been well demonstrated for the uncoupling protein 2 (UCP-2) in the same experimental model used in this study [7].

During steatosis, hepatocytes are overloaded with free fatty acids (FFA), but the liver does not enlarge indefinitely; the hepatocytes reach a new energetic steady state, whereby the increased hepatic FFA uptake and synthesis are compensated by an increased hepatic removal of fatty acids [14].

Carnitine palmitoyl transferase-I (CPT-I), the mitochondrial gateway for fatty acid entry into the matrix, is the main controller of the hepatic mitochondrial β-oxidation flux [15]. In the liver, CPT-I exerts approximately 80% of control under physiological conditions [16]. The impairment in mitochondrial fatty acid oxidation due to down-regulation of hepatic CPT-I is a crucial event in the pathogenesis of hepatic steatosis [17]. To date, the limited number of studies on CPT-I activity during NAFLD did not provide univocal results. In a rodent model of NASH, a significant reduction of CPT-I activity was observed [18] whereas CPT-I activity was found not altered in NASH patients [6]. A remarkable decrease in the expression of the CPT-I gene was instead reported in patients affected by NAFLD [19], [20].

The aim of the present study was to investigate whether mitochondrial CPT-I activity and then fatty acid oxidation efficiencyis affected during MCD-induced steatohepatitis.

In our experiments, a noticeable reduction of CPT-I activity, both in isolated mitochondria and in permeabilized hepatocytes associated to a decreased fatty acid β-oxidation, measured in isolated hepatocytes, was detected in MCD-diet fed rats compared to controls. In the first group of animals an overexpression of CPT-I gene together with a high level of CPT-I protein oxidation was revealed. Our hypothesis is that a posttranslational alteration of CPT-I, occurring during steatohepatitis could be, at least in part, responsible for the reduced mitochondrial fatty acid β-oxidation efficiency.

## Methods

### Animals and experimental design

Adult male Wistar rats (350–400 g) (Harlan, San Pietro al Natisone, Udine, Italy), were caged individually in a temperature and light controlled environment with free access to food and water. Our rats received care in compliance with Italian law (art 4 and 5 of D.L. 116/92). Animals were randomly assigned to the experimental (MCD) or to the control group. MCD-diet fed rats (MCD rats) were fed a high fat/Methionine and Choline Deficient Diet. Control rats consumed the same diet but sufficient in DL-methionine (3 g/kg) and choline bitartrate (2 g/kg) [4]. Diets were purchased from Mucedolas. r.l., Settimo Milanese, Milan, Italy and their compositions have been previously reported [21]. At the end of 4 weeks of diet administration, 5–8 rats of both groups were anesthetized by intraperitoneal injection of 100 mg/kg ketamine and 2.5 mg/kg acepromazine and then sacrificed, their livers removed and weighed. Liver samples were frozen in liquid nitrogen until biochemical analyses were performed.

**Figure 1:**
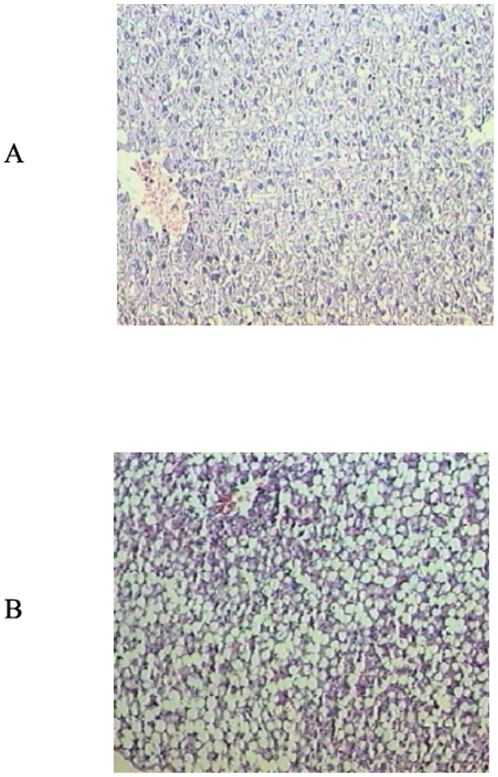
Histological analysis of liver specimens stained with haematoxylin and eosin (H&E) from controls (A) and rats fed the MCD diet (B). Liver of rats fed the MCD diet shows steatohepatitis characterized by panacinar steatosis predominantly as macrovescicular fat, together with lobular inflammation (H&E ×100).

Steatohepatitis was confirmed by histopathological analysis of liver slices (Fig. 1) and serum alanine aminotransferase (ALT) and aspartate aminotransferase (AST) activity assays (Table 1) (Sigma-Aldrich, St. Louis, MO, USA).

**Table 1:** Anthropometric parameters, glucose and lipid content in plasma and liver of rats fed either a control or a methionine-choline deficient diet.

		CTRL	MCD	P
**Body weight (gr)**	d 0	405.2±12.1	399.0±23.0	n.s.
	d 28	419.7±34.9	339.4±32.5	<0.01
**Liver weight (gr)**		12.9±2.4	16.2±1.6	<0.05
**Liver/body weight**		0.031±0.002	0.053±0.004	<0.005
**Serum glucose (mg/dL)**	d 0	189.8±35.1	190.1±37.6	n.s.
	d 28	199.8±38.4	128.8±36.2	<0.05
**Serum triacylglycerols (mg/dL)**	d 0	310.8±89.1	318.1±98.3	n.s.
	d 28	328.7±110.8	38.3±8.8	<0.001
**Serum cholesterol (mg/dL)**	d 0	57.6±11.5	55.8±12.8	n.s.
	d 28	56.8±13.4	28.1±7.5	<0.005
**Serum phospholipids (mg/dL)**	d 0	208.1±24.1	210.1±18.3	n.s.
	d 28	207.7±22.3	93.5±16.6	<0.001
**Serum ALT(U/L)**		39.5±3.9	225.3±70.4	<0.005
**Serum AST(U/L)**		97.5±12.4	163.0±36.8	<0.05
**Hepatic phospholipids(nmol/mg prot)**		54.1±8.3	79.0±7.2	<0.005
**Hepatic triacylglycerols(nmol/mg prot)**		50.5±8.9	253.7±31.4	<0.001
**Hepatic cholesterol (nmol/mg prot)**		12.8±3.0	268.5±50.1	<0.001
**Hepatic glycogen (nmol/mg prot)**		517.5±90.3	268.5±50.1	<0.001

Rats were fed a methionine-choline deficient (MCD) or a control (CTRL) diet for 28 days. Data, expressed as means ± SD of five experiments for each group, are referred at the beginning of the study (d0) and after 4 weeks (d28). Statistical differences were assessed using unpaired t-test assuming variance homogeneity.

### Biochemical analysis

Glucose (GOD-POD method), phospholipids (enzymatic colorimetric method), triacylglycerols (TAG, Triglycerides/GB) and total cholesterol (CHOD-PAP method) in serum and liver were determined as in [22], with test kit from Futura System (Rome, Italy) as specified in parentheses.

### Mitochondria isolation

Liver was removed and rapidly processed for preparation of mitochondria as previously reported [7]. To assess the functional integrity of isolated mitochondria, we measured mitochondrial respiration rate by a Clark-type oxygen electrode adding succinate as substrate. Then, mitochondrial respiration rate in the presence of ADP (state 3 of mitochondrial respiration) and after ADP consumption (state 4) was measured. Mitochondria were used for the experiments when the respiratory control index (state 3/state 4) was >4. Mitochondria protein concentration was determined using the Lowry micromethod kit (Sigma-Aldrich, St. Louis, MO, USA).

### Isolation of hepatocytes

Hepatocytes were isolated by the collagenase perfusion method described in [23]. In order to minimize glycogenolysis, 20 mM glucose was added to the perfusion buffer and to all buffers subsequently employed in the experiments [23]. Hepatocyte suspensions were incubated in Krebs-Henseleit bicarbonate buffer (pH 7.4) (KH buffer) supplemented with 10 mM glucose. Incubations (4–6 mg of cellular protein/ml) were carried out in 25 ml Erlenmeyer flasks in a metabolic shaker (85 oscillations per min) at 37°C in a total volume of 2 ml, under an atmosphere of O_2_/CO_2_ (19∶ 1 v/v).

### Enzymatic determinations

CPT-I activity was assayed in rat liver mitochondria as the incorporation of radiolabelled carnitine into acylcarnitine according to Priore et al. [23]. CPT activity insensitive to 100 µmol/L malonyl-CoA was always subtracted from the CPT activity experimentally determined [23]. The sensitivity of CPT-I to malonyl-CoA inhibition was measured as in [22].

Then, CPT-I activity was determined in digitonin-permeabilized cells using 4 µg/ml digitonin (a concentration chosen after titration with different digitonin concentrations), useful to permeabilize cell membrane, and palmitoyl-CoA as substrate as in [24]. Permeabilized hepatocytes allow to assay rapidly intracellular enzyme activities under more or less physiological conditions and avoiding any possible post-homogenizing modifications which can occur in subcellular fractionation [24].

The activity of 3-hydroxy-acyl-CoA dehydrogenase (3-HAD) was determined spectrophotometrically, in freeze-thawed liver homogenates, according to Giudetti et al. [22].

The activity of acetyl-CoA carboxylase (ACC) was assayed in cytosolic fractions by measuring incorporation of [^14^C]NaHCO_3_ into malonyl-CoA [25].

### Rate of palmitate oxidation in isolated hepatocytes

Fatty acid total oxidation was followed, in hepatocyte suspensions, by adding in KH buffer supplemented with 10 mM glucose, [1-^14^C]palmitate bound to BSA in a 5∶1 molar ratio. Rate of [1-^14^C]palmitate oxidation was determined as formation of total oxidation products, i.e. acid-soluble products (ASP), mostly constituted by ketone bodies, and CO_2_ [23]. Briefly, 2 mL of hepatocytes (4–6 mg of cellular protein/ml) were incubated at 37°C in the presence of 0.5 mM albumin-bound [1-^14^C]palmitate (0.1 Ci/mol). After 20 min, reaction was stopped by the addition of 0.3 ml of 2 M perchloric acid (reactions proceeded at a linear rate up to 45 min). At the same time, 0.15 ml of benzethonium hydroxide (1 M in methanol) was injected in a center well containing filter paper. Samples were allowed to equilibrate for an additional hour at 4°C, and the center well (with the CO_2_ fixed as bicarbonate) was transferred into vials for radioactivity counting. The cell precipitate was spun down, and supernatants were washed three times with light petroleum ether. ASP were subsequently extracted from the samples as in [23]. Total oxidation products were determined as the sum of CO_2_ plus ASP.

### Immunoprecipitation and Western blotting

Mitochondrial proteins (500 µg) isolated from fresh liver tissue were immunoprecipitated at 4 C° overnight with 1 µg anti-CPT-I antibody (rabbit polyclonal CPT-I-L2-A from Alpha Diagnostic, San Antonio, Texas, USA) and 50 µl protein G-agarose beads, using the Protein G Immunoprecipitation Kit (IP-50; Sigma–Aldrich). The immunoprecipitated proteins were loaded onto 12% SDS-PAGE gel and transferred to nitrocellulose membrane for immunoblot analysis with anti-CPT-I antibody (1∶2000). The membrane was then stripped by Restore Stripping Buffer (#21059; Pierce, Rockford, Illinois, USA) for 30 min, washed three times and incubated overnight with anti-HNE antibody (1∶1000; Alpha Diagnostic, San Antonio, Texas, USA). Secondary antibodies were conjugated with horseradish peroxidase and immunoblots detected by VersaDoc Image System (Bio-Rad Laboratories, Hercules, California, USA) as previously reported [7].

To measure ACC protein level, 25 µg of total cytosolic proteins were loaded onto a 7% SDS-polyacrylamide gel. After electrophoresis, the proteins were transferred onto a nitrocellulose membrane. Resulting blot was incubated with ACC antibody (rabbit polyclonal ACC, from Millipore, Billerica, MA, USA) at a dilution of 1∶4000 at room temperature for 2 h, then membrane was incubated for 1 h with secondary horseradish peroxidase-conjugated IgGs (dilution, 1∶5000). Signals were detected by enhanced chemiluminescence. For signal normalization, porin or α-tubulin were used.

### Isolation of RNA from rat liver and Real-Time qPCR analysis

Total RNA from rat liver was isolated using the SV Total RNA Isolation System kit (Promega Italia srl, Milan, Italy), following manufacturer's instructions. RT reaction (20 µl) was carried out using 5 µg of total RNA, 100 ng of random hexamers and 200 U SuperScript™ III RNase H-Reverse transcriptase (Invitrogen srl, San Giuliano Milanese, Milan, Italy). Quantitative gene expression analysis was performed (SmartCycler System, Cepheid) using SYBR Green technology (Celbio, Milan, Italy) and 18S rRNA, for normalization. The primers used for real time PCR analysis were the following:

CPT-I RATFOR 5′-CACTGGCCGAATGTCAAG-3′
CPT-I RATREV 5′-CACGGCTGGATGTTTGCA-3′
18S RATFOR 5′-GTTGGTTTTCGGAACTGAGGC-3′
18S RATREV 5′-GTCGGCATCGTTTATGGTCG-3′

### Measurement of mitochondrial HNE-protein adducts

Liver mitochondria fluorescent adducts formed between 4-Hydroxy-2-Nonenal (HNE) and proteins were monitored by spectrofluorimetry as previously reported [7].

### Fatty acid analysis

To analyze fatty acids, liver mitochondria were saponified with ethanolic KOH for 2 h at 90°C. Fatty acids were extracted [25] and their corresponding methyl esters (FAMEs) were prepared by trans-esterification with 17% methanolic boron trifluoride (BF_3_) at 65°C for 30 min. FAMEs were then analyzed by gas-liquid chromatography [25]. Peak identification was performed by using known standards and relative quantitation was automatically carried out by peak integration.

### Statistical analysis

Data are expressed as means ± standard deviations (SD). The unpaired *t* test was used to assess the significance of differences between means. In all instances P<0.05 was taken as the lowest level of significance. The package GraphPad Prism 4 for Windows (GraphPad Software Inc., San Diego, CA, USA) was used to perform all the statistical analyses.

## Results

### Anthropometric parameters

MCD rats displayed significant weight loss after 28 days with respect to control, although liver weight increased (Table 1). As a consequence, liver/body weight ratio augmented in MCD rats (Table 1). Both these alterations could be taken into account for the onset of steatosis.

### Lipid and glucose content of serum and liver

Serum glucose was significantly reduced after 28 days of the MCD diet administration; a large depletion of serum TAG was also observed in MCD with respect to control rats (Table 1). In the same way, serum concentrations of cholesterol and phospholipids were significantly lowered in the MCD rats when compared to control. Moreover, serum transaminase (ALT and AST) levels were significantly higher in MCD than control rats, indicating an hepatic damage, confirmed also by the histopathological analysis of liver slices (Fig. 1).

TAG and cholesterol levels increased several times in the liver of MCD as compared to control rats (Table 1). Conversely, hepatic glycogen was decreased by about 53% in steatotic liver compared to control (Table 1). Taken together, these data show that the MCD diet markedly modifies liver lipid profile leading to serum lipid modifications.

### CPT-I activity in isolated mitochondria

**Figure 2:**
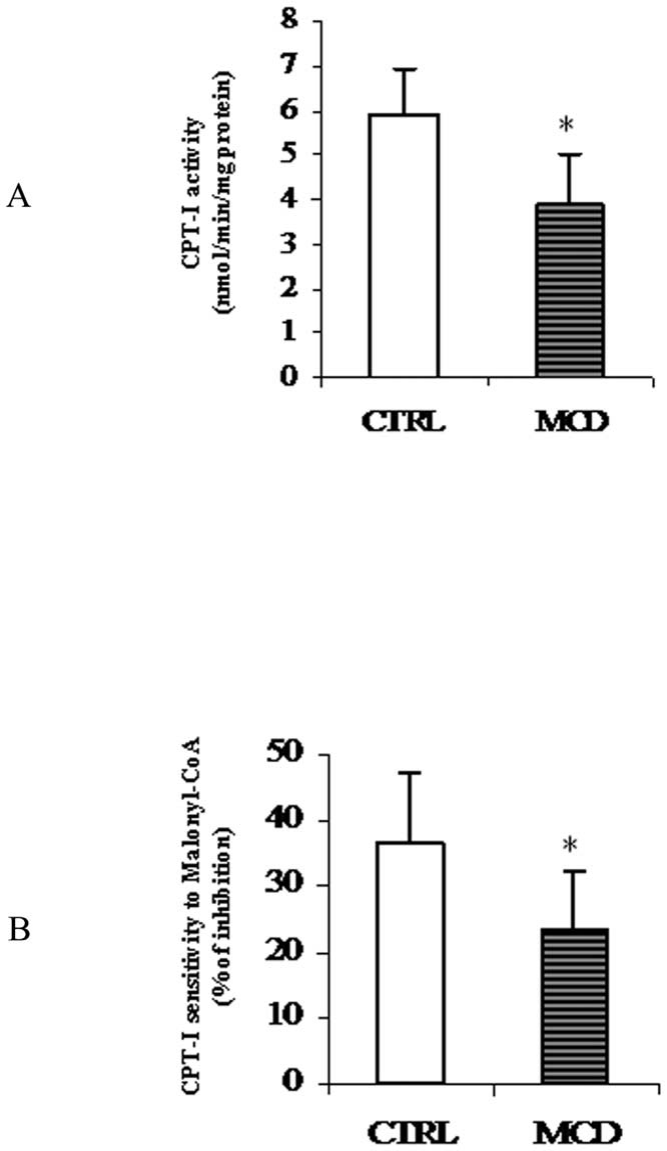
Carnitine palmitoyl transferase-I (CPT-I) activity and sensitivity to malonyl-CoA in isolated mitochondria. Activity of CPT-I (A) and its sensitivity to malonyl-CoA (B) was measured in isolated liver mitochondria from both control (CTRL) and methionine-choline deficient (MCD) diet fed rats. CPT-I activity is reported as nmoles [^14^C]carnitine incorporated into acylcarnitine/min/mg protein. CPT-I sensitivity to malonyl-CoA was expressed as percentage of inhibition to 10 µM malonyl-CoA. Data are expressed as means ± SD of five experiments for each group. Statistical differences were assessed using unpaired t-test assuming variance homogeneity. *Significantly different from the control.

Hepatic fatty acid oxidation was studied using different approaches. Firstly the activity of CPT-I was measured in mitochondria isolated from control and steatotic liver. CPT-I activity was significantly reduced (∼33%) in mitochondria of MCD rats when compared to controls (Fig. 2A). The process of hepatic fatty acid oxidation is controlled not only by CPT-I specific activity but also by its sensitivity to the level of malonyl-CoA [16] produced by the first step of fatty acid synthesis catalyzed by ACC. Moreover, CPT-I sensitivity to malonyl-CoA is regulated by changes in lipid composition that are localized to specific membrane microdomains [26]. Thus, hepatic CPT-I sensitivity to malonyl-CoA inhibition was determined.

Interestingly, in steatotic liver the reduced CPT-I activity was accompanied by a decrease (∼36%) in sensitivity to malonyl-CoA (Fig. 2B).

**Figure 3:**
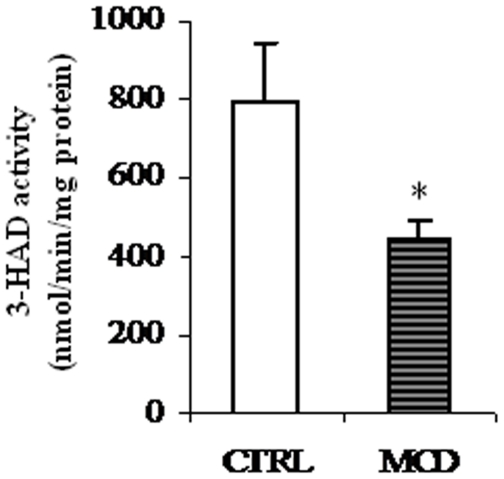
Effects of the methionine-choline deficient (MCD) diet on 3-hydroxy-acyl-CoA dehydrogenase (3-HAD) activity. Activity of 3-HAD of control (CTRL) and methionine-choline deficient (MCD) diet fed rats was measured spectrophotometrically as described in “Methods”. Data are expressed as means ± SD of five experiments for each group. Statistical differences were assessed using unpaired t-test assuming variance homogeneity. *Significantly different from the control.

Once in mitochondrial matrix, fatty acids are oxidized by the β-oxidation pathway. A remarkable reduction in the activity of 3-hydroxy-acyl-CoA dehydrogenase (3-HAD), key enzyme of the mitochondrial matrix β-oxidation process, was observed in liver mitochondria of MCD rats as compared to controls (Fig. 3).

### Assay of ACC activity

As above reported, short-term control of CPT-I activity involves inhibition by malonyl-CoA produced by ACC. Because the latter enzyme is a key regulatory site of *de novo* fatty acid synthesis, malonyl-CoA inhibition of CPT-I allows an elegant explanation for the coordinate control of the partition of hepatic fatty acids into esterification and oxidation [16].

**Figure 4:**
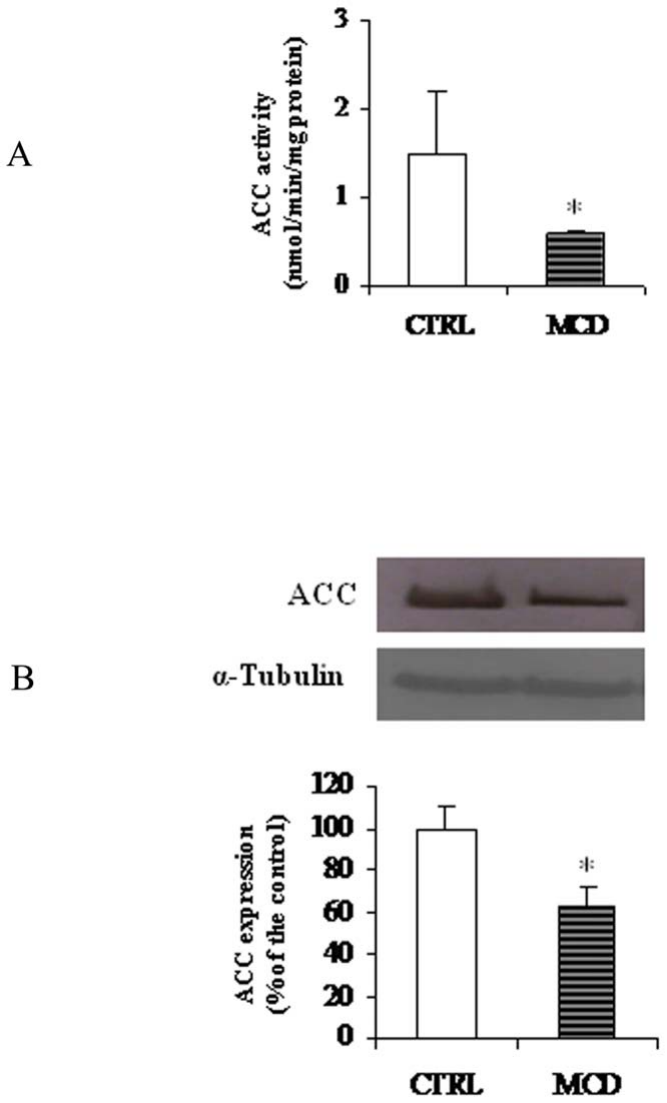
Activity and protein level of acetyl-CoA carboxylase (ACC). ACC activity (A) and protein level (B) in control (CTRL) and methionine-choline deficient (MCD) diet fed rats were measured as reported in “Methods”. ACC activity is expressed as nmoles [^14^C]NaHCO_3_ incorporated into malonyl-CoA/min/mg protein. α-Tubulin was used for signal normalization. Data are expressed as means ± SD of five experiments for each group. Statistical differences were assessed using unpaired t-test assuming variance homogeneity. *Significantly different from the control.

Specific activity of ACC was significantly lower in MCD than in control rats (0.6±0.04 vs 1.5±0.71 nmol/min/mg protein, P<0.001) (Fig. 4A). ACC protein content, assayed by Western blot analysis, decreased in parallel with the activity (Fig. 4B).

### CPT-I assay in permeabilyzed hepatocytes

It has been demonstrated that the activity of liver CPT-I is affected by extramitochondrial cell components that are lost on isolation of mitochondria [24]. Permeabilizing cells with digitonin opens the possibility of investigating intracellular enzymes in a more or less natural environment. This experimental tool is particularly useful when interactions exist among cytosolic enzymes, such as ACC, with organelle associated enzymes, if their active centre faces the cytosolic space as is the case of CPT-I [24]. A noticeable sensitivity of the latter enzyme to the malonyl-CoA level was observed in permeabilized hepatocytes [24], thus indicating that this approach is very suitable for investigating the ACC/malonyl-CoA/CPT-I system.

At this point, it is important to underline that CPT-I activity, assayed in situ by using digitonin-permeabilized hepatocytes, showed an inhibition (0.19±0.01 nmol/min/10^6^ cells of MCD rats vs 0.26±0.02 nmol/min/10^6^ cells of controls; n = 3, P<0.05) of the same extent found in isolated mitochondria.

### In situ fatty acid oxidation assay

**Figure 5:**
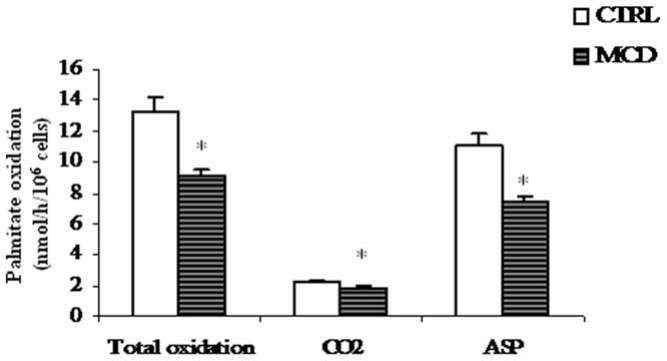
Palmitate oxidation by rat hepatocytes. Hepatocytes were incubated with [1-^14^C]palmitate. Total fatty acid oxidation was obtained as the sum of CO_2_ and total acid-soluble products (ASP). Results, expressed as [1-^14^C]palmitate into product/h/10^6^ cells, correspond to means ± SD of three different experiments. *Significantly different from the control.

Next, total rate of fatty acid oxidation was investigated following the oxidation of [1-^14^C] palmitate added to hepatocyte suspensions. Acetyl-CoA, produced by mitochondrial β-oxidation of fatty acids, can enter the citric acid cycle to be degraded to CO_2_ for energy production if the supply of oxaloacetate is sufficient or, alternatively, can give rise to ketone bodies (i.e. acid soluble products, ASP). Therefore, the total oxidation of fatty acids is a summation of the metabolism towards ASP and CO_2_. We found that either CO_2_ production and particularly ASP formation were significantly lower in hepatocytes from MCD rats than in those from controls (Fig. 5). Consequently, total [1-^14^C]palmitate oxidation was reduced (∼30%) in hepatocytes from MCD animals with respect to controls (9.1±0.6 nmol/h/10^6^ cells of MCD vs 13.3±0.9 nmol/h/10^6^ cells of control; n = 3, P<0.05). Note that the percentage of inhibition of fatty acid oxidation in hepatocytes from MCD rats well correlates with the decrease of CPT-I activity measured in isolated mitochondria (see Fig. 2A).

### CPT-I protein and mRNA expression

**Figure 6:**
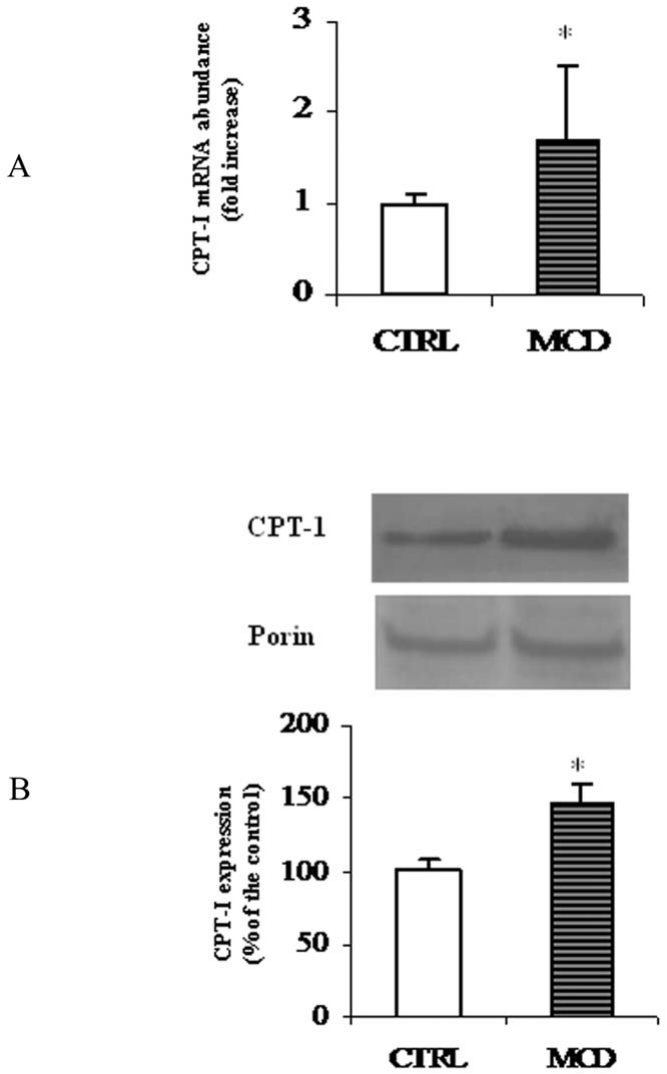
mRNA and protein levels of rat liver CPT-I. The histogram (A) represents CPT-I mRNA abundance determined using RT-quantitative PCR and expressed as relative amount (18s rRNA as a reference) in liver from control (CTRL) and methionine-choline deficient (MCD) diet fed rats (A). Data are shown as mean values ± SD for the CPT-I gene relative to an arbitrary value of 1, which was assigned to the expression level in control animals. Western blot analysis of CPT-I in liver from CTRL and MCD rats (B) was performed as reported in “Methods”. Porin was used for signal normalization. The content of CPT-I was quantified by densitometric analysis. Data are mean values ± SD of five experiments for each group. *Significantly different from the control.

To investigate the molecular mechanism underlying the MCD-induced decrease of CPT-I activity, CPT-I mRNA and protein levels were quantified by Real Time PCR and Western blot analysis, respectively. To this end, total RNA was isolated from liver of MCD and control rats and Real-time qPCR analysis was performed. As shown in Fig. 6A an increase of approx. 70% of CPT-I mRNA abundance was observed in MCD rats when compared tocontrols.

Next, CPT-I expression was investigated by Western blot analysis (Fig. 6B). As shown in the figure, densitometric analysis indicated that liver mitochondrial CPT-I protein level in MCD rats was considerably higher (approx. 40%)than in the control ones. Porin, the mitochondrial outer membrane channel, was used as a control protein, because it has been reported that its expression is not affected by dietary treatment [27].

### Protein oxidation assay

Accumulating evidence suggests that mitochondrial dysfunction participates in NASH pathogenesis, since it impairs fatty liver homeostasis and induces overproduction of ROS [14]. These latter, in turn, trigger lipid peroxidation, cytokines release and cell death. Aldehydes, such as HNE, are major end products of lipid peroxidation and, by constituting adducts with proteins, can accumulate in tissues under oxidative stress [14].

Previous reports have demonstrated HNE-protein adduct formation in several experimental models of NASH [28], [29]. We have also demonstrated the occurrence of the adducts in the MCD model [7].

**Figure 7:**
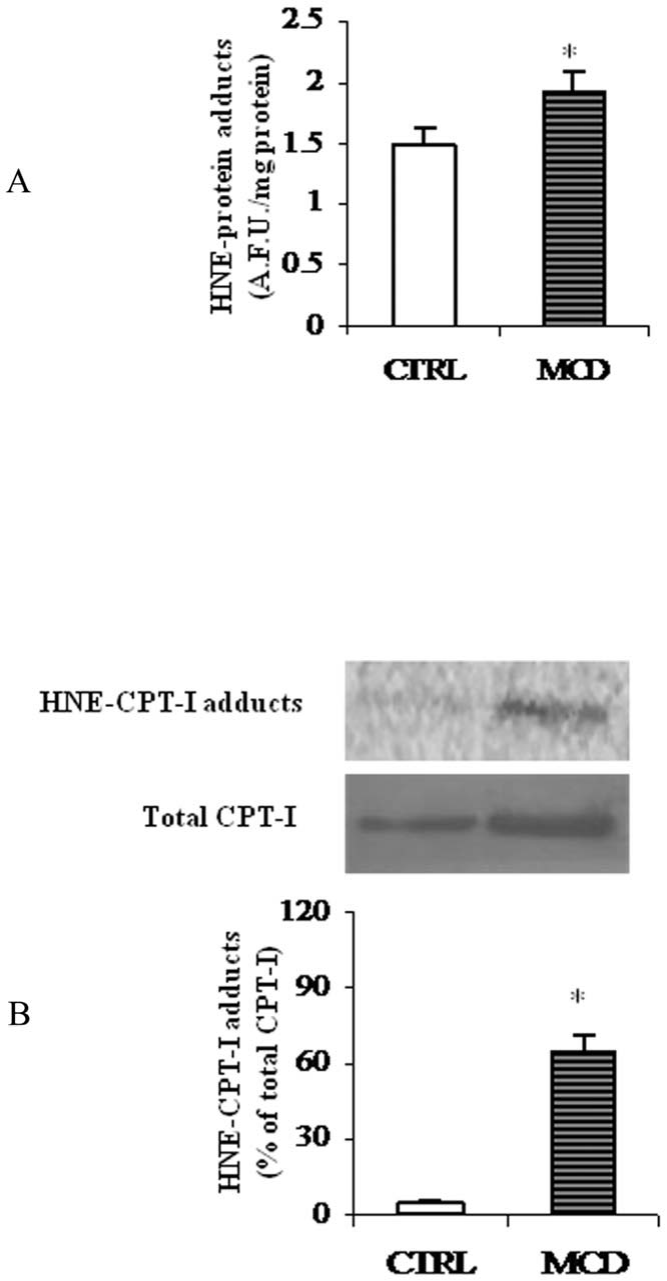
HNE-protein adducts in liver mitochondria. (A) Levels of HNE-protein adducts in liver mitochondria from methionine-choline deficient (MCD) diet and control rats (CTRL) were measured by fluorimetric analysis and evaluated in terms of arbitrary fluorescent units (A.F.U.) at 355 nm excitation and 460 nm emission. Data are expressed as means ± SD of five experiments for each group. (B) Western blot analysis was performed to reveal HNE-CPT-I adducts from CTRL and MCD rats. Signals were quantified by densitometric analysis and expressed as % of the total CPT-I protein content measured in mitochondria from CTRL and MCD rats. Statistical differences were assessed using unpaired t-test assuming variance homogeneity. *Significantly different from the control.

In the present work HNE-protein adduct ssignificantly increased in the liver of MCD rats as compared to controls (Fig. 7A).

To test the hypothesis that impairment in CPT-I activity could be related to protein oxidation, mitochondrial proteins were immunoprecipitated using anti-CPT-I antibody and then revealed by using anti-HNE-protein adduct antibody [7]. As reported in Fig. 7B, a signal consistent with HNE-CPT-I adduction was observed almost exclusively in mitochondria from MCD rats(65% of the total CPT-I protein content).

### Fatty acid composition of hepatic mitochondrial membranes

Fatty acid composition of mitochondrial membranes from liver of control and MCD rats was then analyzed. Even though the ratio of saturated to unsaturated fatty acids in MCD rats was unaltered, a significant decrease in linoleic and arachidonic acids, both of the n-6 series, occurs together with an increase in the docosapentaenoic and docosahexaenoic acids (Table 2). In agreement with Rizki et al. [30] which reported an inhibitory effect of MCD diet on the stearoyl-CoA desaturase-1 activity in mouse liver, a reduction of oleic and palmitoleic acid levels was also observed in mitochondria of MCD animals, when compared to controls.

**Table 2:** Fatty acid composition of rat liver mitochondria.

Fatty acid		CTRL	MCD	P
		mol%	mol%	
Myristic	C 14∶0	0.29±0.07	0.18±0.08	<0.05
Palmitic	C 16∶0	17.66±1.07	18.11±0.86	n.s.
Palmitoleic	C 16∶1	0.55±0.20	0.28±0.16	<0.05
Stearic	C 18∶0	19.78±2.38	22.97±2.16	n.s.
Oleic	C 18∶1	8.72±1.34	6.91±0.41	<0.05
Linoleic	C 18∶2	21.09±1.42	18.75±1.72	<0.05
Arachidonic	C 20∶4	25.54±1.57	21.50±0.74	<0.001
Eicosapentaenoic	C 20∶5	1.02±0.48	0.87±0.15	n.s.
Docosapentaenoic	C 22∶5	0.32±0.07	1.51±0.34	<0.0001
Docosahexaenoic	C 22∶6	3.50±0.50	9.44±1.15	<0.0001
Σsat		36.81±3.00	40.86±2.45	n.s.
Σunsat		60.55±2.52	58.41±3.78	n.s.
Σsat/Σunsat		0.62±0.08	0.71±0.09	n.s.

Rats were fed the methionine-choline deficient (MCD) diet or a control (CTRL) diet for 28 days. Σsat = sum of saturated fatty acids ; Σunsat = sum of unsaturated fatty acids. Data are expressed as means ± SD of five experiments for each group. Statistical differences were assessed using unpaired t-test assuming variance homogeneity.

## Discussion

Steatosis, induced in rodents by an MCD diet, presents pathological and biochemical similarities with human steatohepatitis, although it does not induce insulin resistance [31]. The absence in the diet of choline and methionine, which are precursors for the biosynthesis of phosphatidylcholine, an essential component of plasma lipoproteins, interferes with the synthesis and secretion of the very low density lipoproteins (VLDL). In these conditions the strong reduction in the transport of TAG by VLDL out of the hepatocytes leads to steatosis [32].

Impaired hepatic oxidation and removal of fatty acids may also contribute to the raise in lipid content in liver of MCD mice [30].

Our data confirm increased lipid accumulation, mainly as TAG, in the liver of MCD rats together with a reduction in the serum lipid level (see Table 1) probably due to a phospholipid-related defect in hepatic secretion [30].

The present study shows that MCD diet, administered to rats for 4 weeks, decreases CPT-I activity both in isolated mitochondria and in permeabilized hepatocytes thus producing pronounced effects on hepatic lipid metabolism that go beyond the simply inhibition of TAG secretion.

The role of liver mitochondrial CPT-I, key enzyme of fatty acid oxidation pathway, in steatosis onset and/or progression is still matter of debate.

To date, few and controversial results on CPT-I activity and/or expression modifications occurring during NASH development, have been reported. Perez-Carreras et al. [6] did not find any difference in the liver CPT-I activity of NASH patients as compared to controls. However, Nakamuta et al. [19] evaluating the expression of fatty acid metabolism-related genes in NASH patients, found a remarkable increase of ACC mRNA abundance, but a decrease of CPT-I expression thus suggesting a posttranscriptional mechanism for CPT-I regulation In a rodent nutritional model of NASH a significant reduction of CPT-I activity, together with a decreased CPT-I sensitivity to malonyl-CoA, was observed. This reduction was ascribed to the nitration of the protein [18].

Such diversity of results may be related to differences in the grade and the stage of NASH, in the model adopted and in the methods used to measure CPT-I activity and expression.

Permeabilization of hepatocyte membrane with digitonin might offer a closer physiological environment to study mitochondrial processes under precisely controlled conditions *in situ*, where mitochondrial interaction with intracellular structures, is largely preserved [24]. In the present studywe measured a reduced activity of CPT-Iin digitonin-permeabilizedhepatocytes from MCD rats. The lower CPT-I activity measured in treated animals well correlate with the decreased activity of the key mitochondrial matrix enzyme 3-HAD (Fig. 3) and with the low rateof total fatty acid oxidation measured in hepatocyte suspensions (Fig. 5). Such inhibition may contribute totheaccumulation of fatty acids that enter the pathway of esterification determining afurther increase of hepatic TAG amount, as indeed found (Table 1).

In the present work we also report,in MCD rats, anover-expression of CPT-Igene as indicated by the increment of bothCPT-I mRNA abundance and protein levels. To explain why, despite a higher CPT-I expression, we measured a low CPT-I activity during NASH, when compared to controls, we assume that CPT-I over-expression could occur into the liver during the onset of steatosis, to counteract the elevated fatty acid level as reported by [30], and some other posttranslational modifications could, subsequently, reverse the effect. The reduction of CPT-I sensitivity to malonyl-CoA together with the inhibition of ACC activity and expression we measured, could further support the idea that a compensatory mechanism, acting through a regulation of the ACC/malonyl-CoA/CPT-I system [33], is triggered in the first phase of NASH, when liver is still steatotic, to facilitate fatty acid oxidation.

At this point it must be underlined that increased mitochondrial fatty acid β-oxidation, insteatotic liver,is a widely recognized event [14]. Fromenty et al [11] claim that, to avoid an indefinite enlargement,steatotic liver reaches a new metabolic steady state characterized by high rates of mitochondrial fatty acid β-oxidation [11].

The process by which steatosis falls into steatohepatitis is a topic of great interest. Studies have recently provided persuasive evidences that oxidative stress occurs in several different animal models of steatohepatitis and in patients with NASH [34]. In both cases the degree of lipid peroxidation correlates with the severity of steatosis [34]. ROS-mediated lipid peroxidation is an attractive candidate for a central role in the pathogenesis of NASH.

HNE, a very reactive electrophilic aldehydes created by peroxidation of n-6 fatty acids, such as arachidonic and linoleic acid esters, can covalently attach to proteins by forming adducts with cysteine, lysine, or histidine residues, thereby damaging the protein structure. Such damage may affect the protein function and its own catabolism [34], [35]. HNE has been readily detected in the mitochondria of hepatocytes in patients with NASH [36]. In our experiments a reduced level of liver mitochondria n-6 fatty acids (see Table 2), probably due to their increased peroxidation, was detected in MCD rats. Since HNE is the main product of n-6 fatty acid oxidation, we tested whether HNE may bind CPT-I during steatosis and we observed HNE-CPT-I adducts were almost exclusively present in the liver of MCD rats (see Fig. 7). Note that, in the same animal model we have previously reported the formation of an adduct between HNE and UCP-2 and a consequent modification of the activity of the bounded protein [7]. Therefore, CPT-I protein oxidation, causing a decrease in the amount of active protein, could account for the reduced activity measured in our experiments despite the higher level of CPT-I expression.

In conclusion, the present work shows that during NASH, induced by MCD dietary treatment, some products of oxidative stress, such as HNE, may bind and modify proteins as CPT-I. The aldheyde-protein interaction could interfere with the fatty acid transport into mitochondria and therefore with their oxidation, leading to an impairment in lipid removal.

To our knowledge, our data represent the first evidence reporting covalent modification occurring on CPT-I during steatohepatitis. However, the fully understanding of the relationship between oxidative stress and development of steatohepatitis requires further elucidations.
